# Case Report: Hydronephrosis and nephrogenic adenoma secondary to Stevens-Johnson syndrome

**DOI:** 10.3389/fphar.2025.1624432

**Published:** 2025-08-15

**Authors:** Chuan Wang, Yidong Huang

**Affiliations:** Department of Pediatric Surgery, West China Hospital, Sichuan University, Chengdu, China

**Keywords:** Stevens-Johnson syndrome, hydronephrosis, nephrogenic adenoma, adverse reaction, complication

## Abstract

**Introduction:**

Stevens–Johnson syndrome is a rare, severe cutaneous adverse reaction. There is a lack of literature reporting the complications of the bladder and ureter caused by Stevens–Johnson syndrome. In this case, we describe a 10-year-old boy with hydronephrosis and nephrogenic adenoma secondary to Stevens–Johnson syndrome.

**Case description:**

A boy was admitted to our hospital after a CT scan revealed bilateral hydronephrosis. He had been diagnosed with Stevens–Johnson syndrome earlier. Ureteroscopy and bilateral retrograde ureteral stenting were performed. After the guidewires were introduced, a mass of mucosal debris gushed from the ureteral orifices. Biopsy findings of the mucosal lesions were consistent with nephrogenic adenoma. Two months later, ultrasonography revealed worsening hydronephrosis. The double-J stents were removed. Follow-up ultrasonography showed an improvement in the hydronephrosis.

**Conclusion:**

Stevens–Johnson syndrome is a life-threatening mucocutaneous disorder. Ureteral obstruction and hydronephrosis can be caused by mucosal debris in patients with Stevens–Johnson syndrome, which may contribute to and exacerbate renal dysfunction. Nephrogenic adenoma can occur to patient with Stevens–Johnson syndrome as a result of the proliferation of implanted renal tubular cells. Routine ultrasonography should be conducted to monitor the urinary system.

## Introduction

Stevens–Johnson syndrome (SJS) is a rare, severe cutaneous adverse reaction, commonly triggered by medications, and is characterized by widespread epidermal necrosis of the skin and mucosa, with significant associated morbidity and mortality (Owen and Jones, 2021; Schneider and Cohen, 2017). While this condition typically affects ocular and cutaneous surfaces, it may also involve mucosal membranes including those of the gastrointestinal, respiratory, and genitourinary systems (Owen and Jones, 2021). The majority of genitourinary complications of SJS have been documented well, including balanitis, urethral erosions, urethral strictures, glomerulonephritis and chronic renal dysfunction (Saeed et al., 2016). Reports of bladder and ureter complications remain notably scarce. Herein, we present the case of a 10-year-old boy with hydronephrosis and nephrogenic adenoma secondary to SJS and discuss the management of hydronephrosis complicated by SJS.

## Case description

**FIGURE 1:**
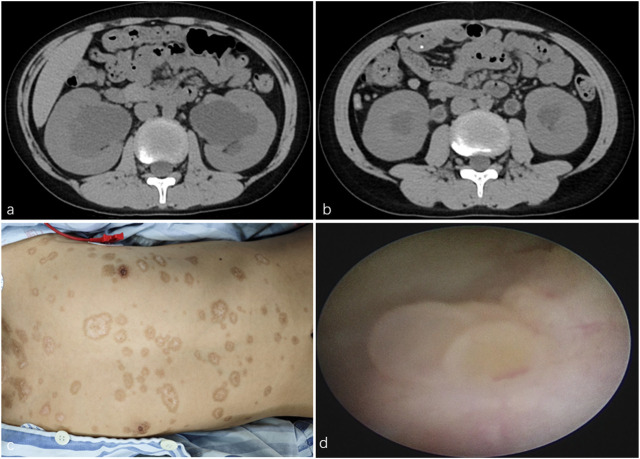
**(a)** CT scan revealed bilateral hydronephrosis. **(b)** CT scan revealed bilateral hydroureter. **(c)** Physical examination found scattered hypopigmented patches with centripetal depigmentation, mainly on the trunk. **(d)** Ureteroscopy found scattered follicular mucosal lesions in the bladder.

A 10-year-old boy was admitted to our hospital after a CT scan revealed bilateral hydronephrosis (Figure 1a) with hydroureter (Figure 1b). He had been diagnosed with SJS 5 months earlier, with an initial chief complaint of fever and a concomitant skin rash. During outpatient follow-up after completion of SJS treatment, a CT scan revealed bilateral hydronephrosis with hydroureter. Other than this, the boy had an unremarkable medical and family history. Physical examination revealed bilateral symblepharon and scattered hypopigmented patches with centripetal depigmentation, mainly on the trunk (Figure 1c). Laboratory tests indicated normal levels of serum creatinine and blood urea. Pulmonary function test revealed severe impairment.

Given his history of SJS, the possibility of bilateral ureteral obstruction caused by mucosal debris was considered. Ureteroscopy and bilateral retrograde ureteral stenting were performed. Ureteroscopy found scattered follicular mucosal lesions in the bladder (Figure 1d) and mucosal impairment in the urethra and ureters. After the guidewires were introduced into the ureters, a mass of mucosal debris and sludge gushed from the ureteral orifices. Fr 4.7 double-J stents were placed. Histopathological examination of the mucosal biopsy tissue revealed small hollow tubules and focal papillary structures. Immunohistochemical staining demonstrated positive expression for PAX8, AMACR, and CK7, while showing negative results for CEA, CDX2, CR, and MIB-1. Biopsy findings were consistent with nephrogenic adenoma (NA).

**FIGURE 2:**
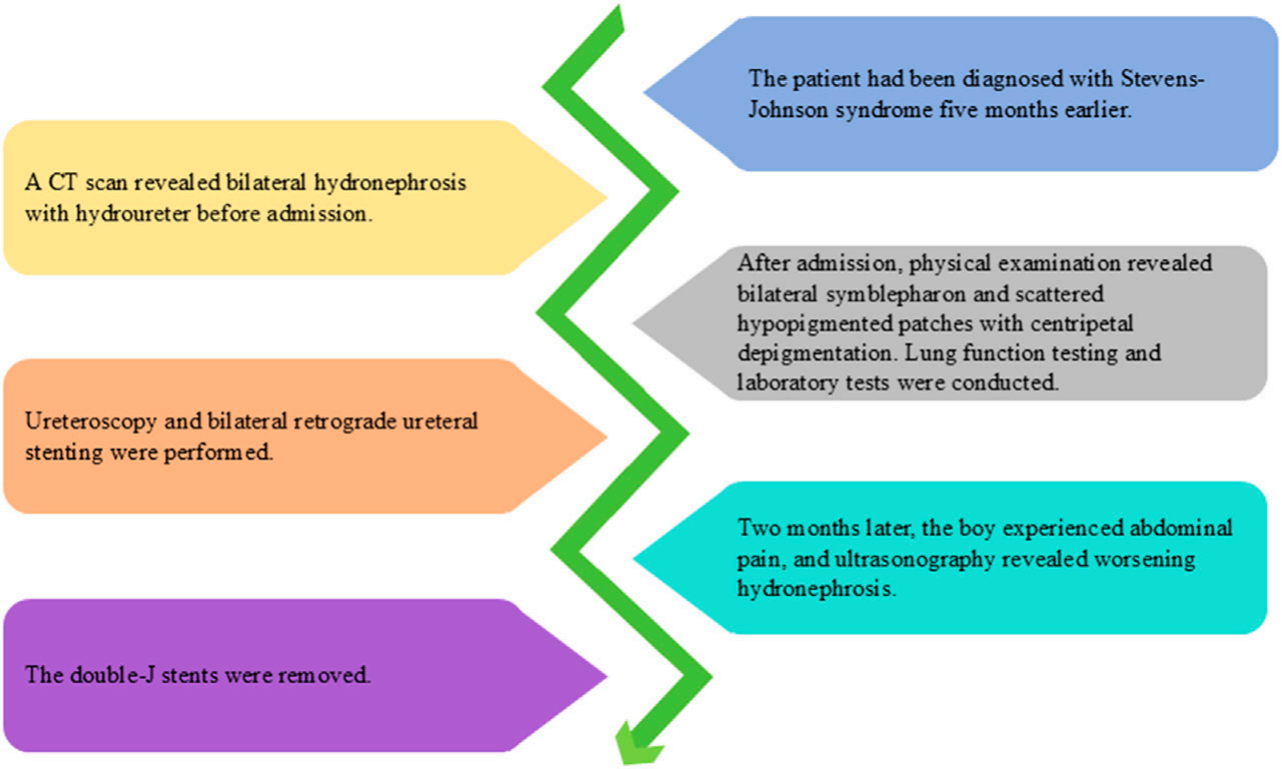
Timeline diagram of disease and treatment course.

Ultrasonography showed a decrease in hydronephrosis 1 week after surgery. However, 2 months later, the boy experienced abdominal pain, and ultrasonography revealed worsening hydronephrosis. The double-J stents were removed (Figure 2), and we found the stents were obstructed by mucosal debris. After the stents were removed, patient’s abdominal pain was relieved. Follow-up ultrasonography showed a reduction in hydronephrosis. Subsequently, the patient underwent ultrasound re-examinations every 2 months for 1 year, with no observed worsening of hydronephrosis in any of the follow-ups.

## Discussion

SJS is a life-threatening mucocutaneous disorder. In this case, the impaired pulmonary function was caused by airway obstruction secondary to epidermal necrosis of the respiratory tract mucosa. Although lower urinary tract complications are frequently reported, complications affecting the upper urinary tract are rarely documented. To our knowledge, there was only one case report that documented ureteral obstruction and hydronephrosis in a patient with SJS (Baccaro et al., 2014). In that case, the patient developed ureteral perforation caused by mucosal impairment and ureteral obstruction. Similarly, ureteral obstruction in the patient was caused by mucosal debris and double-J stents were placed. Near half of patients with SJS develop acute renal dysfunction, which is commonly attributed to acute tubular necrosis or prerenal azotemia (Saeed et al., 2016; Letko et al., 2005). Based on cases of ureteral obstruction by mucosal debris, we believe that ureteral obstruction may contribute to and exacerbate renal dysfunction. Therefore, routine ultrasonography should be performed to monitor the urinary system in patients with SJS. The long-term urological complications associated with SJS have not been well characterized in current literature, and consequently there is no consensus regarding optimal follow-up duration. We recommend a minimum of 1 year of follow-up to monitor for potential late-onset urological sequelae.

NAs are rare, benign lesions of the urinary tract, with most found in the bladder (Mazal et al., 2002). Initially thought to be a metaplasia of the urothelium in response to chronic inflammation or injury, NAs are now recognized as the result of proliferation of implanted renal tubular cells along the urinary tract (Mazal et al., 2002). We speculate that the NA in this case was caused by acute renal tubular injury trigger by SJS. Although NAs are typically solitary, this case presented with multifocal lesions. NA can mimic malignant tumors, such as clear cell adenocarcinoma, papillary urothelial carcinoma, and nested variant of urothelial carcinoma. In our case, the diagnosis of NA was confirmed through pathological examination. When bladder lesions are found in a patient with a history of SJS, NA should be considered to prevent misdiagnosis. Additionally, some reports suggest that NA may undergo malignant transformation (Hartmann et al., 2006; Hungerhuber et al., 2008). Therefore, ultrasonography should be routinely performed during follow-up to monitor for NA.

In conclusion, ureteral obstruction and hydronephrosis in patients with SJS can be caused by mucosal debris, which may contribute to and exacerbate renal dysfunction. NA can develop in patients with SJS as a result of the proliferation of implanted renal tubular cells. Routine ultrasonography should be conducted to monitor the urinary system in patients with SJS.

## Data Availability

The original contributions presented in the study are included in the article/supplementary material, further inquiries can be directed to the corresponding author.
